# Scalable production and application of *Pichia pastoris* whole cell catalysts expressing human cytochrome P450 2C9

**DOI:** 10.1186/s12934-021-01577-4

**Published:** 2021-04-26

**Authors:** Javier Garrigós-Martínez, Astrid Weninger, José Luis Montesinos-Seguí, Christian Schmid, Francisco Valero, Claudia Rinnofner, Anton Glieder, Xavier Garcia-Ortega

**Affiliations:** 1 Department of Chemical, Biological and Environmental Engineering, School of Engineering, Universitat Autònoma de Barcelona, 08193 Bellaterra (Cerdanyola del Vallès), Spain; 2 Institute of Molecular Biotechnology, Graz University of Technology, Petersgasse 14, 8010 Graz, Austria; 3 Bisy GmbH, Wuenschendorf 292, 8200 Hofstaetten/Raab, Austria; 4 Austrian Centre of Industrial Biotechnology (ACIB), Petersgasse 14, 8010 Graz, Austria

**Keywords:** Bidirectional promoters, Human CYP2C9, Ibuprofen, *Pichia pastoris*, Bioprocess optimisation, Whole cell biocatalyst

## Abstract

**Background:**

Currently, the numerous and versatile applications in pharmaceutical and chemical industry make the recombinant production of cytochrome P450 enzymes (CYPs) of great biotechnological interest. Accelerating the drug development process by simple, quick and scalable access of human drug metabolites is key for efficient and targeted drug development in response to new and sometimes unexpected medical challenges and needs. However, due its biochemical complexity, scalable human CYP (hCYP) production and their application in preparative biotransformations was still in its infancy.

**Results:**

A scalable bioprocess for fine-tuned co-expression of hCYP2C9 and its essential complementary human cytochrome P450 reductase (hCPR) in the yeast *Pichia pastoris* (*Komagataella phaffii*) is presented. High-throughput screening (HTS) of a transformant library employing a set of diverse bidirectional expression systems with different regulation patterns and a fluorimetric assay was used in order to fine-tune hCYP2C9 and hCPR co-expression, and to identify best expressing clonal variants. The bioprocess development for scalable and reliable whole cell biocatalyst production in bioreactors was carried out based on rational optimization criteria. Among the different alternatives studied, a glycerol carbon-limiting strategy at high *µ* showed highest production rates, while methanol co-addition together with a decrease of *µ* provided the best results in terms of product to biomass yield and whole cell activity. By implementing the mentioned strategies, up to threefold increases in terms of production rates and/or yield could be achieved in comparison with initial tests. Finally, the performance of the whole cell catalysts was demonstrated successfully in biotransformation using ibuprofen as substrate, demonstrating the expected high selectivity of the human enzyme catalyst for 3′hydroxyibuprofen.

**Conclusions:**

For the first time a scalable bioprocess for the production of hCYP2C9 whole cell catalysts was successfully designed and implemented in bioreactor cultures, and as well, further tested in a preparative-scale biotransformation of interest. The catalyst engineering procedure demonstrated the efficiency of the employment of a set of differently regulated bidirectional promoters to identify transformants with most effective membrane-bound hCYP/hCPR co-expression ratios and implies to become a model case for the generation of other *P. pastoris* based catalysts relying on co-expressed enzymes such as other P450 catalysts or enzymes relying on co-expressed enzymes for co-factor regeneration.

**Supplementary Information:**

The online version contains supplementary material available at 10.1186/s12934-021-01577-4.

## Background

In order to avoid adverse drug reactions (ADRs) not only the drug as such, but also the impact of its metabolites on the organism are considered of great importance [1]. From the super-family of P450 enzymes from different origins, Human cytochrome P450s (hCYPs) are a group of heme-containing membrane-associated monooxygenases involved in the oxidation of monoterpenes, saturated fatty acids and alkanes, vitamins, steroids and eicosanoids as well as in the clearance of drugs and xenobiotics in humans [2, 3]. Among the human variants, next to CYP3A4 and CYP2D6, CYP2C9 is one of the most important drug oxidizing enzyme [4]. In particular, CYP2C9 oxidizes numerous drugs such as celecoxib, diclofenac, etodolac, ibuprofen, indomethacin, lornoxicam, mefenamic acid, suprofen, tenoxicam and, additionally, also metabolizes endogenous compounds such as arachidonic acid, linoleic acid, and non-drug xenobiotics [4].

Drugs and drug-metabolite standards are required for toxicological, biological and drug-to-drug interaction studies. Their synthesis is often challenging, since selective hydroxylation of non-activated carbon atoms is difficult to achieve by chemical synthesis. These studies are elucidated by the employment of tissue samples (e.g. human liver homogenate) or microsomal preparations thereof [5–7]. Both are not scalable for preparative synthesis on multi mg scale. Microbial P450 enzymes provide an interesting alternative but frequently do not produce the desired specific products, or undesired mixtures which are different from the product spectrum made by human P450s are obtained [6, 8–10]. Adaptation by protein engineering is possible but in cases of unexpected arising needs for new drugs such as arising epidemic outbreaks, but such approach is usually too slow. Therefore, based on their versatile and changing applications in drug metabolite synthesis, there is an increasing interest and high demand for hCYPs in both pharmaceutical and chemical industries, especially when applicable on a preparative scale level [3, 11]. In this sense, biotransformations performed with purified enzymes are hardly feasible on a large scale, since hCYPs usually present low efficiency and stability [11, 12]. To some extend this is also related to the fact the hCYP’s are membrane associated, so they can be considered as membrane proteins. However, selective access of some substrates to the active site is known to rely on the interface between enzyme and the ER membrane [13, 14]_,_ expression of membrane associated hP450s seems preferable compared to soluble expression of truncated P450s. Furthermore, to be biologically active, CYPs require an electron transport system which provides the electrons to the CYPs for oxygen activation and substrate oxidation [15]. hCYPs rely on the presence of a cytochrome P450 reductase (CPR), which is needed for electron transfer from the co-factor NAD(P)H. The co-expression of hCPR often showed to be more effective than taking advantage of the host’s own CPR(s) [7].

During the last years, to enable industrial biotransformations that require high conversion rates, easy handling and low costs, the recombinant production of CYP enzymes has been intensively investigated [6, 10, 12, 16–19]. Some relevant publications have reported several cases for the recombinant expression of human P450s in different cell factories [3, 20]. Specifically, successful expression of hCYP2C9 has been reported for insect cells [21], *Escherichia coli* [22] and *Schizosaccharomyces pombe* [23]. Thus, this enzyme was used for the preparation of milligram amounts of the 4′-hydroxy metabolite of diclofenac using whole cells or isolated membranes [22]. The use of yeast expression systems as whole cell biocatalysts was described by Neunzig et al*.*, who demonstrated the applicability of an ibuprofen conversion process using fission yeast for the co-expression of hCYP2C9 and CPR [24, 25], and efficient and selective 4′-hydroxy diclofenac was synthesized on g-scale by the Bureik lab [23]. In a comparative study Geier et al*.* highlighted the methylotrophic yeast *Pichia pastoris,* recently re-classified as *Komagataella phaffii*, as a preferable cell factory for whole cell bioconversions using human CYP2D6 [7]. Due to those experiences and the broad availability and establishment of the *P. pastoris* expression system in thousands of labs, we were interested to study the reliability, scalability and performance of *P. pastoris* whole cell catalysts expressing hCYP2C9.

*Pichia pastoris* is currently considered as an efficient alternative for recombinant protein production combining the simplicity of bacterial expression systems with some essential features of higher eukaryotic hosts such as mammalian cells. Most of membrane proteins (MPs) of interest such as CYPs often come from eukaryotic organisms, thus bacterial expression systems often fail to express such genes to get biologically active and corrected folded enzymes. This fact has been described to be due to the lack capacity to perform post-translational modifications (e.g. disulphide bridge formation or glycosylation), the inefficient secretory pathways needed to direct the protein to the membrane, as well as to the differences in lipid bilayer composition [26, 27] and availability of different intracellular compartments. On the other hand, mammalian expression systems are able to produce complex proteins, and especially MPs [27]. However, their cell growth is significantly slower and more expensive compared to microbial cells. Thus, on the basis of its high efficiency, wide availability and cost-effectiveness, *P. pastoris* has become one of the most frequently used host systems for the production recombinant proteins, including membrane-anchored and soluble proteins—either intra or extracellular [27]. Usually, most published studies focused on the recombinant expression of MPs address issues related to protein degradation, ER folding problems and post-translational modifications in order to increase final product titers, since it may have a relevant impact on its production [28, 29]. However, to the date, there has not been reported any study that assess the impact of operational strategies in the recombinant production of MPs targeted to be anchored in the cell membrane. So, as a highly relevant factor to design efficient scalable processes, this work aimed to determine the interrelation between the specific production rate (*q*_*p*_) and the specific growth rate (*µ*) of the culture for active hP450 catalyst production and biotransformations in bioreactors. This relationship is called production kinetics, and can be considered the key to design optimal bioprocess strategies for recombinant protein production (RPP) [30–36].

The aim of this work was to demonstrate a model case for the production of highly active and selective CYP2C9/CPR whole cell biocatalysts using the simple, widely available and efficient expression system *P. pastoris.* It takes into consideration the essential scalability required for catalyst manufacturing by the design and implementation of a reliable and optimized cultivation process for whole cell catalyst production. Therefore, in a complementary approach, the catalyst expression bioprocess was optimized by evaluating different co-expression ratios of hCYP and hCPR using a bidirectional and functionally balanced expression system and also further combined with a rationally designed bioprocess at bench-top bioreactor scale taking into consideration the relevant impact of pH, specific growth rate and the use of methanol as co-substrate on whole cell biocatalyst production. The efficiency of such produced whole cell catalysts was demonstrated by preparative scale biotransformation (0.5 L) of ibuprofen to produce the main hCYP2C9 ibuprofen metabolite 3-hydroxyibuprofen.

## Results and discussion

### Strain generation

Expression systems based on engineered bidirectional promoters offer a simple and quick solution to enable multi-gene co-expression in which the expression of each gene can be optimally tuned towards to achieve the desired objective [37]. To fully exploit the potential of this bidirectional expression system, a test set of 7 alternative promoters with diverse regulation patterns was used in different co-orientations to drive the expression of the full-length genes: *CYP2C9* (AL359672) including its hydrophobic N-terminal sequence and redox partner h*CPR* (accession AAH34277.1). In contrast to self-sufficient soluble bacterial P450s, here the CPR is covalently linked to the heme domain of the P450 catalyst, thus different co-expression levels and regulatory profiles were evaluated in order to achieve the best performance of the whole cell biocatalyst requiring two separate functionally expressed peptide chains, anchored to the membrane in optimal orientation and proximity to its redox partner. This study included the strong methanol inducible promoters P_*DAS1*_ and P_*DAS2*_ (and their shortened variants P_*DAS1-552*_ and P_*DAS2-699*_), as well as the promoters P_*PDC*_ and P_*PDF*_, two derepressed promoters in which the recombinant expression is activated upon glucose/glycerol depletion, and additionally it is further inducible in presence of methanol. In earlier studies using GFP as a model protein, P_*DAS1*_ presented a strong methanol-inducible performance, reaching at least half of the expression level of the commonly used *AOX1* promoter, while P_*DAS2*_ even outperformed P_*AOX1*_ expression [38]. On the other hand, the expression of the P_*PDC*_ promoter (native catalase gene promoter) is repressed in the presence of glucose or glycerol and derepressed upon the depletion of these carbon sources. Its expression can further be induced with methanol and remarkably also with oleic acid [39]. Besides the P_*PDC*_, an orthologous promoter, i.e. P_*PDF*_, which exhibits a similar regulation profile, was employed to regulate gene transcription. These promoters, which allow different levels of induction, enable to some extend to uncouple recombinant protein expression and cell growth for *P. pastoris* and therefore become interesting tools for bioprocess optimization.

Earlier studies reported the efficiency of CYP2D6 in *P. pastoris* whole cells, which was detectable by an spectroscopy method based on carbon monoxide (CO) difference [7]. However, highest CYP levels determined by CO-spectroscopy could not be correlated to highest conversions levels when using whole cells. Working with whole cells, their conversion efficiencies are influenced by electron and substrate supply [7], and it also affected by the complex interactions of multiple parameters (e.g. CYP/CPR ratio, membrane permeability, NADH production/regeneration or expression level of ROS degrading enzymes between among others) [40]. Thus, this work is focused on the achievement of active whole cells with optimized CYP/CPR ratios rather than a maximization of P450 as determined by carbon monoxide spectroscopy following a previously described bidirectional expression strategy [37].

**Fig. 1:**
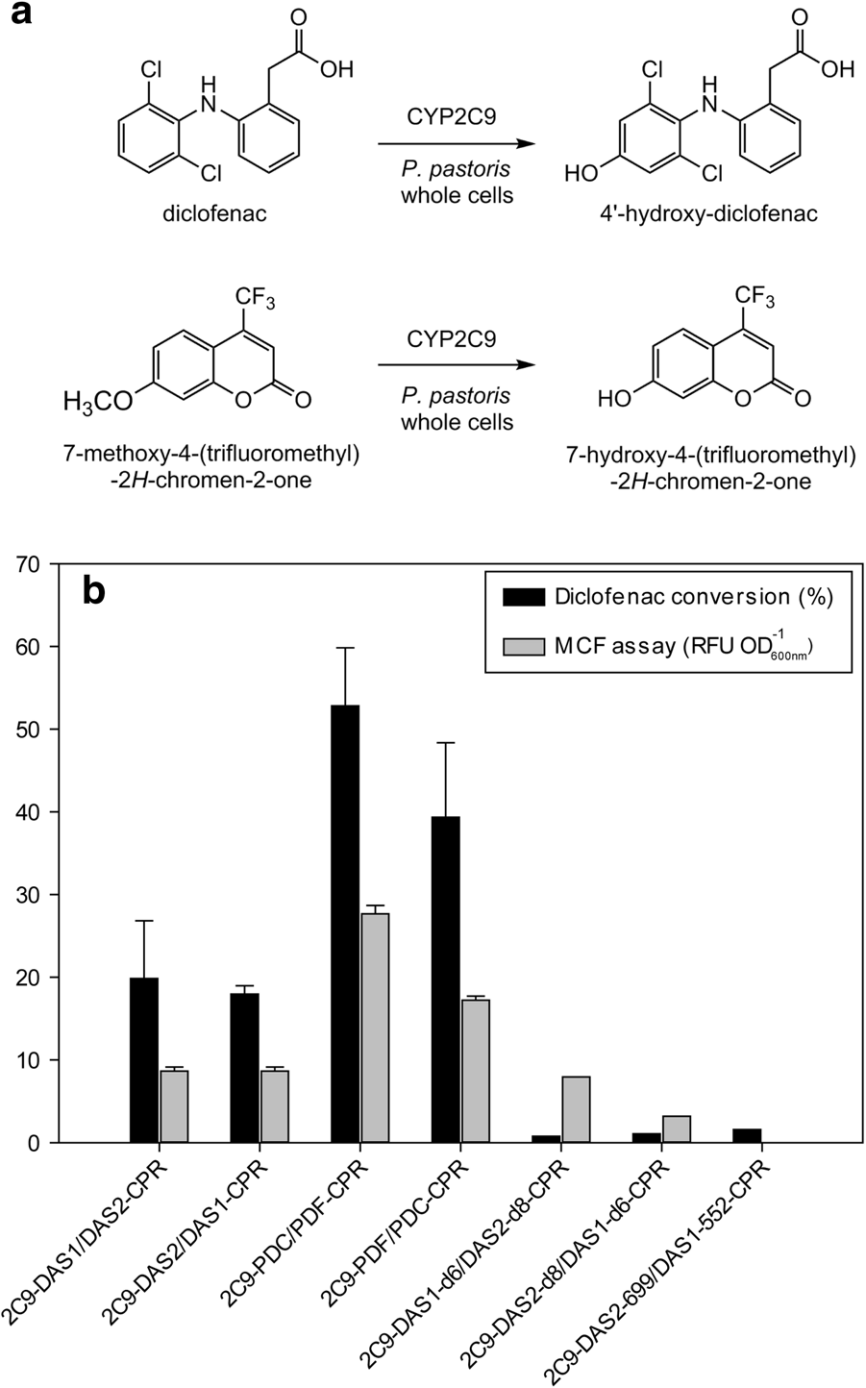
**a** Diclofenac and 7-methoxy-4-(trifluoromethyl)-2*H*-chromen-2-one (MCF) oxidation reactions catalysed by *P. pastoris* CYP2C9/CPR whole cell biocatalyst. **b** Screening of producer clones based on bidirectional expression system using different promoter variants towards to identify the most active CYP2C9/CPR whole cells

For the identification of the catalyst with the highest activity, two alternative whole cell bioconversions assays suitable for High Throughput Screening (HTS) were used [41]. One based on diclofenac conversion, and the other one based on the fluorescence of the product 7-hydroxy-4-(trifluoromethyl)-2*H*-chromen-2-one (HFC). Both analysis methods were used for comparing the best clone’s performance of each construct, as shown in Fig. 1. The qualitative behaviour of both screening methods was rather similar, construct 2C9-PDC/PDF-CPR showed the best conversion (53%) in the diclofenac-based assay (2 mM). The same construct with inverted promoter orientation 2C9-*PDF*/*PDC*-CPR, also presented good substrate conversion reaching 39%, while all other constructs showed only 20% conversion of diclofenac, or even lower. Similar results were obtained using the alternative fluorometric MCF-based assay, demonstrating the applicability of this simple assay for the identification of cells with highest P450 activity in simple 96-deep well plate assays with low cell densities cultures. As for the diclofenac screening, the expression system built on P_*PDF*_/P_*PDC*_ combination provided the best results, reaching a maximum value of 28 RFU per OD_600_. However, due to the low sample numbers the activity of samples obtained from bench-top fermenters, was directly determined by HPLC for the drug target substrate diclofenac.

The applied screenings identified transformants with a P_*PDF*_ promoter for CYP expression and P_*PDC*_ for the CPR (clones 2C9-PDC/PDF-CPR) as most promising for the further bioprocess development in bench-top fermenters.

### Production of *P. pastoris* CYP2C9/CPR whole cell biocatalyst in bioreactor cultures

For the very first time, CYP2C9/CPR expressing *P. pastoris* cells were cultivated successfully in bioreactor. Previous unpublished studies in our lab prior to this cultivation optimization demonstrated unexpected difficulties in the reproduction of biocatalyst activities observed in shake flasks after cultivation in methanol induced 5 L bioreactor cultures. Thus, it was considered interesting to compare hCYP2C9/hCPR expressing cells cultivated in either shake flask and bioreactor regarding their ability to convert diclofenac. As presented in Additional file 1: Fig. S1, the conversion rates obtained per mg of biomass dry cell weight (DCW) for catalysts generated in this study were rather similar. After confirmation that cells obtained from bench-top fermenters displayed similar diclofenac oxidation activities than cells from shake flask, a thorough study of hCYP2C9/hCPR whole cell production was carried out including chemostat cultivation and bioprocess optimization in fed-batch cultures.

**Fig. 2:**
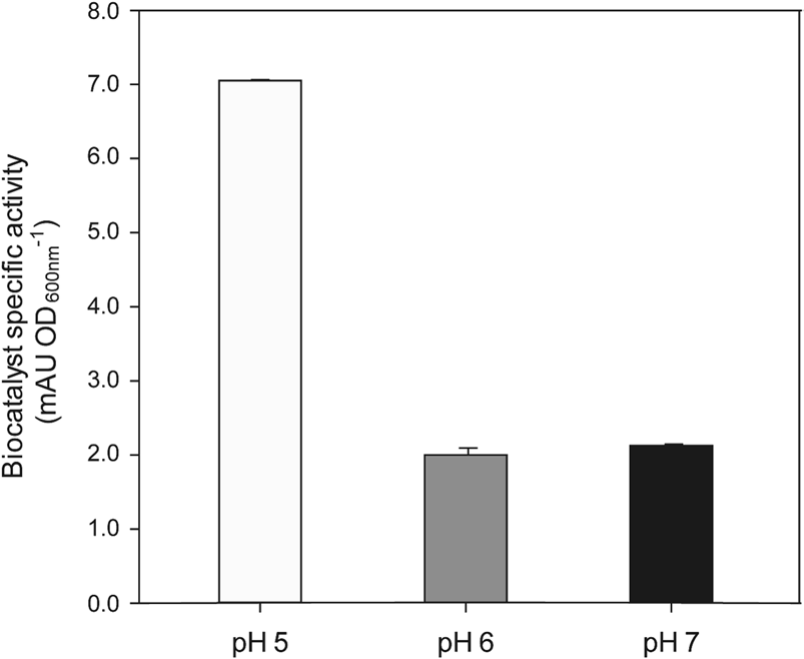
Effect of culture pH for the production of *P. pastoris* CYP2C9/CPR whole cells biocatalyst. Chemostat cultivations were carried out, setting the dilution rate to 0.10 h^−1^. Error bars represent the activity assay standard deviation determined by triplicates

Among the physical–chemical parameters that could affect the bioprocess performance, pH was identified to have significant impact. The control and adjustment of pH can hardly be controlled or adapted in 96 well plate cultures, with high buffer concentrations as the only way to keep at least a desired pH constant. Previous experiments in micro scale deep well plate cultures showed best results with a starting pH of 8, which most likely drop to lower values during cultivation. Thus, the effect of different pH values (pH 5–7) on the production of CYP2C9/CPR whole cells in chemostat cultivations at intermediate *D* = 0.10 h^−1^ was assessed systematically. Working in steady state conditions for a constant specific growth rate (*µ*), provides a robust and reliable cultivation system that allows studying the effect of a specific operational parameter while keeping the others constant. Activity tests showed that the best condition was pH = 5, obtaining a specific enzyme activity up to threefold higher than in the other conditions tested (Fig. 2). Consequently, further fed-batch (FB) cultivations were always carried out at pH = 5.

#### Biomass growth characterization in fed-batch cultures

Since the producer strain identified by screening for best performing transformants was based on the bidirectional expression construct 2C9-PDC/PDF-CPR, the regulation pattern of the used promoters allowed to implement either methanol-induced and/or methanol-free bioprocess strategies or consecutive induction schemes by derepression followed by induction. Therefore, as the current trend of *P. pastoris* bioprocesses is to avoid the use of toxic and flammable methanol due to its operational associated drawbacks, a primary set of methanol-free FB cultures with glycerol as sole carbon source was performed. In these cultures, the effect of the specific growth rate (*µ*) on biocatalyst production was evaluated by performing glycerol-limiting cultures at different nominal *µ* (FBs 1–3). Furthermore, the potential boosting effect of methanol on biocatalyst production was additionally assessed by growing the culture at *µ* = 0.05 h^−1^ on glycerol-limiting conditions and adding methanol as co-substrate either by pulses as the simplest strategy (FB4), or keeping a constant methanol concentration of 3 g L^−1^ (FB5). The last strategy was selected since it was previously reported to be the best condition for *Rhizopus oryzae* lipase production under the control of P_*AOX1*_ promoter [42].

In designed co-substrate cultivations using the Mut^S^ host strain phenotype, it was necessary to maintain a low *µ*—0.05 h^−1^, otherwise methanol utilisation (MUT) genes might be repressed [43, 44]. Consequently, methanol would not be consumed and its impact as promoter inducer might not be properly assessed.

**Fig. 3:**
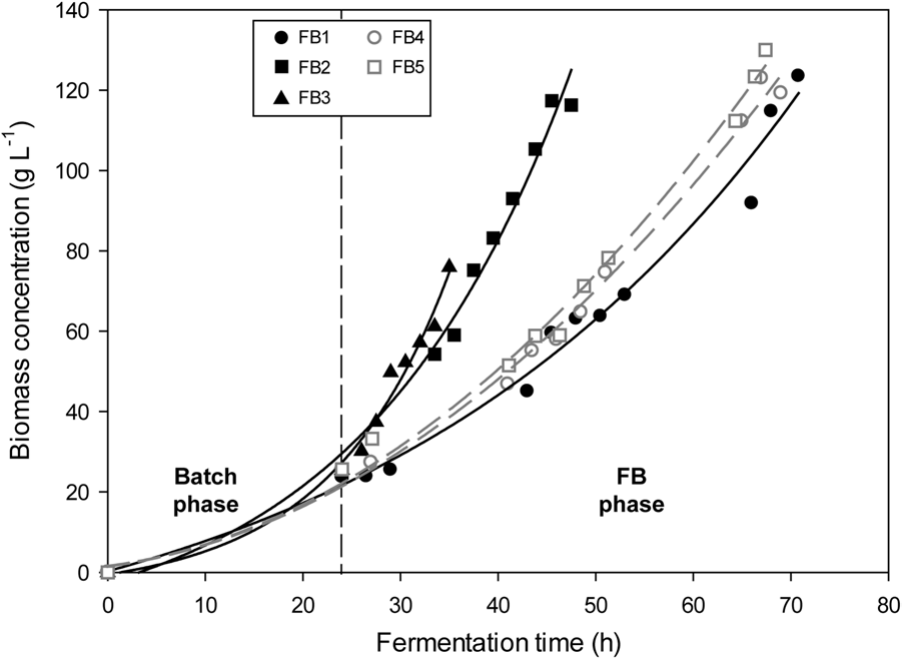
**a** Biomass time-profile of the fed-batch cultures performed. Experiment labels: FB1: *μ* = 0.05 h^−1^; FB2: *μ* = 0.10 h^−1^; FB3: *μ* = 0.15 h^−1^; FB4: *μ* = 0.05 h^−1^ + MetOH pulses; FB5: *μ* = 0.05 h^−1^ + constant MetOH = 3 g L^−1^

Concerning biomass generation, the identified most active transformant (named *P. pastoris* 2C9-PDC/PDF-CPR) was able to grow as expected in all three methanol-free FB cultures (Fig. 3). Although some growth effect might have been expected for such membrane protein overexpression strain, the recombinant expression did not have any significant limiting effect on the cell growth. Most of the cultures were grown up to reach 100 g L^−1^ of DCW. Nevertheless, the fermentation performed at 0.15 h^−1^ (FB1) had to be stopped at a biomass concentration around 80 g L^−1^. At this point, the temperature control could no longer keep constant the temperature at 25 °C due to the high amount of heat generated at high specific growth rate. Anyway, numerous samples were taken throughout the process, which allowed the determination of the production-related macrokinetic parameters needed for its comparison with the rest of FB cultures.

Due to the low biomass-to-methanol yield, *Y*_*X/MetOH*_, and the slow specific methanol uptake rate, *q*_*MetOH*_, of the *P. pastoris* phenotype (Mut^S^ strains), the use of methanol as a co-substrate did not modify the growth curves substantially when comparing with methanol-free processes. Moreover, CYP2C9/CPR production did not alter significantly other relevant cell physiology rates, as indicated by the specific glycerol uptake rate (*q*_*Gly*_) and biomass-to-substrate yield (*Y*_*X/Gly*_) (Table 1). The obtained values were similar to those usually reported as standard for *P. pastoris* strains producing different recombinant proteins [45–47].

**Table 1:** Main process parameters obtained in the different fed-batch cultures performed for the production of active hCYP2C9/hCPR *P. pastoris* whole cell biocatalysts

Run	*Y*_*X/S*_ g_X_ g_S_^−1^	*q*_*Gly*_ g_Gly_ g_X_^−1^ h^−1^	*q*_*MetOH*_ g_MetOH_ g_X_^−1^ h^−1^	Biocatalyst activity AU L^−1^	*q*_*p*_ AU g_X_^−1^ h^−1^	*Y*_*P/X*_ AU g_X_^−1^	*Y*_*P/MetOH*_ AU g_MetOH_^−1^
FB1	0.47	0.102	–	1.78	0.67	13.4	–
FB2	0.51	0.159	–	1.52	0.78	10.4	–
FB3	0.46	0.272	–	1.17	1.71	12.9	–
FB4	0.55	0.084	0.029	3.28	1.15	21.9	39.7
FB5	0.53	0.087	0.023	2.68	1.10	21.1	47.8

µ: specific groth rate, Y_X/S_: Biomass to substrate yield; *q*_*Gly*_: specific glycerol uptake rate; *q*_*MetOH*_: specific methanol uptake rate; *q*_*P*_: specific producrtion rate; *Y*_*P/X*_*:* Product to biomassa yield; *Y*_*P/MetOH*_: Product to methanol yield;

FB1: 0.05 h^−1^; FB2: 0.10 h^−1^; FB3: 0.15 h^−1^; FB4: 0.05 h^−1^ + MetOH pulses; FB5: 0.05 h^−1^ + MetOH constant 3 g L^−1^

#### Production of CYP2C9/CPR in *P. pastoris* whole cells in fed-batch cultures

**Fig. 4:**
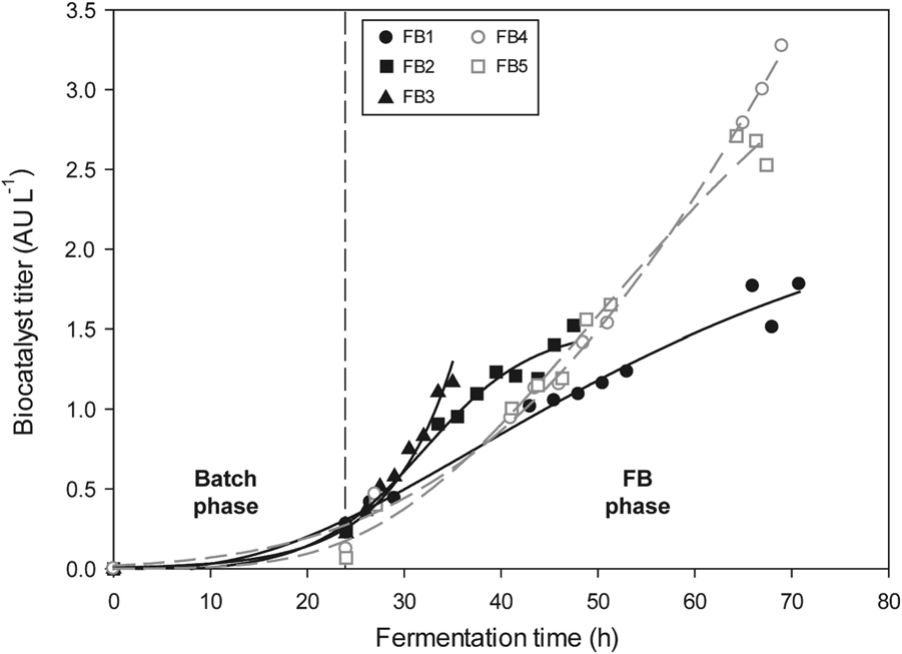
Biocatalyst activity time-profiles obtained in fed-batch cultures 1–5. Experiment labels: FB1: *µ* = 0.05 h^−1^; FB2: *µ* = 0.10 h^−1^; FB3: *µ* = 0.15 h^−1^; FB4: *µ* = 0.05 h^−1^ + MetOH pulses; FB5: *µ* = 0.05 h^−1^ + constant MetOH = 3 g L^−1^

Regarding whole cell biocatalyst activity, the production-time profiles were analysed for the five alternative FB strategies. The obtained results (Fig. 4), demonstrated significant differences in terms of biocatalyst activity between the methanol-free fed-batches cultures (FB 1–3) and those induced with methanol (FB 4–5). In methanol-free processes, a saturation trend was observed in late stage of the profiles at low and mid *µ*—0.05^–1^; 0.1 h^−1^, whereas at the highest µ tested—0.15 h^−1^—always a growth-coupled production profile was observed. On the contrary, the methanol induced processes present an exponential trend up to 3.28 AU L^−1^, thus reaching biocatalyst activity relevantly higher than methanol-free fermentations. Specifically, when further inducing the system with methanol a twofold increase in biocatalyst activity could be observed.

As presented in Fig. 4, a significant but low amount of active biocatalyst was obtained consistently already at the end of the batch phase in all five cultures. This fact is an effect of the derepressed expression in non-limiting glycerol conditions of the promoters used, P_*PDF*_/P_*PDC*_. However, later, at glycerol-limiting conditions the expression was boosted as expected. The similar starting point at the end of the batches carried out demonstrated also the high reproducibility between the different bioreactor runs.

**Fig. 5:**
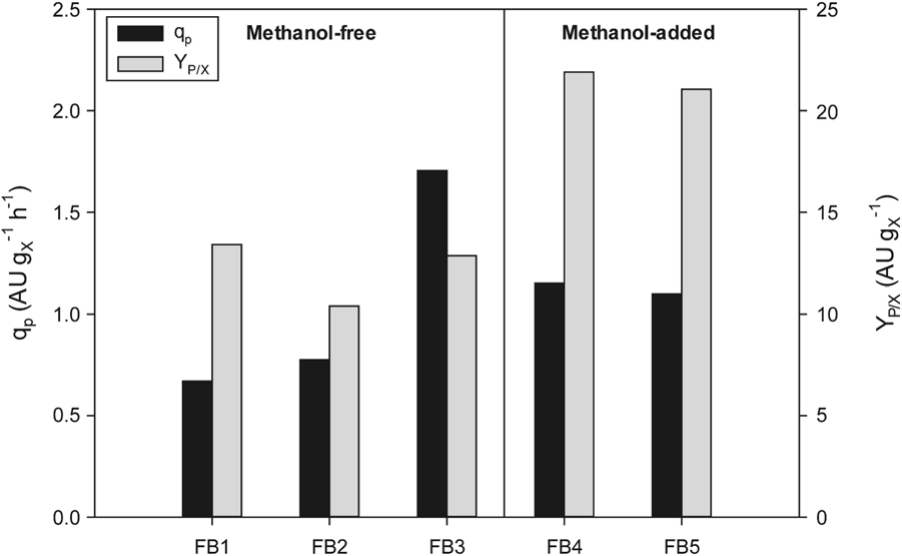
Product-related parameters obtained in fed-batch cultures: specific product generation rate (*q*_*p*_) and product-to-biomass yield (*Y*_*P/X*_). Experiment labels: FB1: *µ* = 0.05 h^−1^; FB2: *µ* = 0.10 h^−1^; FB3: *µ* = 0.15 h^−1^; FB4: *µ* = 0.05 h^−1^ + MetOH pulses; FB5: *µ* = 0.05 h^−1^ + constant MetOH = 3 g L^−1^

It has been widely described that the specific production rate (*q*_*p*_) is importantly affected by *µ* when expressing a heterologous protein regulated by different promoters. In several previous studies was reported that cell growth and protein production were coupled [33, 45, 48, 49]. As depicted in Fig. 5, the *q*_*p*_ in this study was clearly correlated with *µ* within the experimental range tested. Results pointed out a slight increase of *q*_*p*_ when increasing the specific growth rate from 0.05 h^−1^ (FB1) to 0.10 h^−1^ (FB2). However, a bigger increase was found when growing cultures at 0.15 h^−1^, reaching up to a 2.5-fold *q*_*p*_ increase respect to the worst condition. In contrast, the *µ* effect on *Y*_*P/X*_ values might be considered negligible as yield values are rather similar for all three fed-batches, around 11.5 AU g_X_^−1^ (FB 1–3).

With respect to the effect of methanol utilization on biocatalyst activity production, bioreactor cultivations with the same *µ* (FB1, FB4 and FB5) but either adding or not adding methanol were compared. In all the cultures, the use of this co-substrate further induced the protein expression, significantly increasing the production rates. More specifically, it led to *q*_*p*_ improvements of up to 1.72-fold, and up to 1.63-fold of *Y*_*P/X*_. On the one hand, this can be due to the effect that recombinant gene expression is highly induced in presence of methanol with both promoters used, P_*PDF*_ and P_*PDC*_. However, the observed effects might also be attributed to other differences in carbon source dependent systems-level organization, such as for example co-factor regeneration, membrane permeability, or the expression levels of other proteins [40]. As a summary, a comparison of the main process parameters is presented in Table 1. When comparing both methanol-based culture strategies implemented, strikingly, controlling the methanol concentration in the culture broth (FB5) only provided a slight increase of about 20% in terms of active biocatalyst produced per gram of methanol fed (*Y*_*P/MetOH*_) compared to the methanol-pulses based strategy (FB4). Moreover, other product-related parameters—*q*_*p*_, *Y*_*P/X*_—are rather similar when controlling the methanol at a constant concentration. Consequently, since the results obtained are similar, the methanol pulses-based process can be considered as preferable due to its technical simplicity comparing with a cultivation strategy based on a closed-loop methanol control.

In order to maximize the bioprocess efficiency, different optimization criteria based on diverse performance indexes can be considered; among them, biocatalyst activity, productivity and yield are the most often selected. In *P. pastoris* fermentation processes, the culture is often limited by the maximum amount of biomass that can be reached, about 100 g L^−1^ in DCW. Then, the optimization method should aim to maximise the production before reaching this limiting cell concentration. To maximise the product activity (*P*) and yield (*Y*_*P/X*_*)*, the process should be prolonged in time, growing the cells not only at a low *µ*, but also adding methanol as co-substrate, preferably through pulses. In this way, up to 3.28 AU L^−1^ could be achieved, this representing a threefold increase respect to the worst condition. On the contrary, if *q*_*p*_ is selected as the parameter to be optimized, the best operational strategy would be a carbon-limiting fed-batch operation using glycerol as a sole carbon source at high *µ.* Specifically, setting the *µ* at the highest value tested—0.15 h^−1^ (FB3); a threefold increase could be achieved respect to worst conditions.

However, from an application point of view, in order to select the optimal bioprocess strategy to produce an active biocatalyst like the presented in this work, the whole workflow including downstream steps should also be taken into consideration during the optimization process. Since this bioprocess approach uses whole cells, downstream processing can be considered rather simple and not costly. Therefore, the need to reach high biocatalyst activities, which is essential to be able to afford high cost separations, should not be considered as priority. Thus, taking into consideration the different criteria discussed previously, the methanol-free carbon-limiting strategy based on glycerol at highest *µ* should be considered the best alternative that allows maximization of the product generation rates (*q*_*p*_). Despite lower biocatalyst activity levels reached, avoiding the use of methanol would allow to avoid the important methanol-associated operational drawbacks. These relevant drawbacks, including high oxygen demand, heat production, and additional costs related to methanol storage, transportation and handling need to be considered for further scaling steps of the bioprocess [38].

### Preparative scale demonstration

**Fig. 6:**
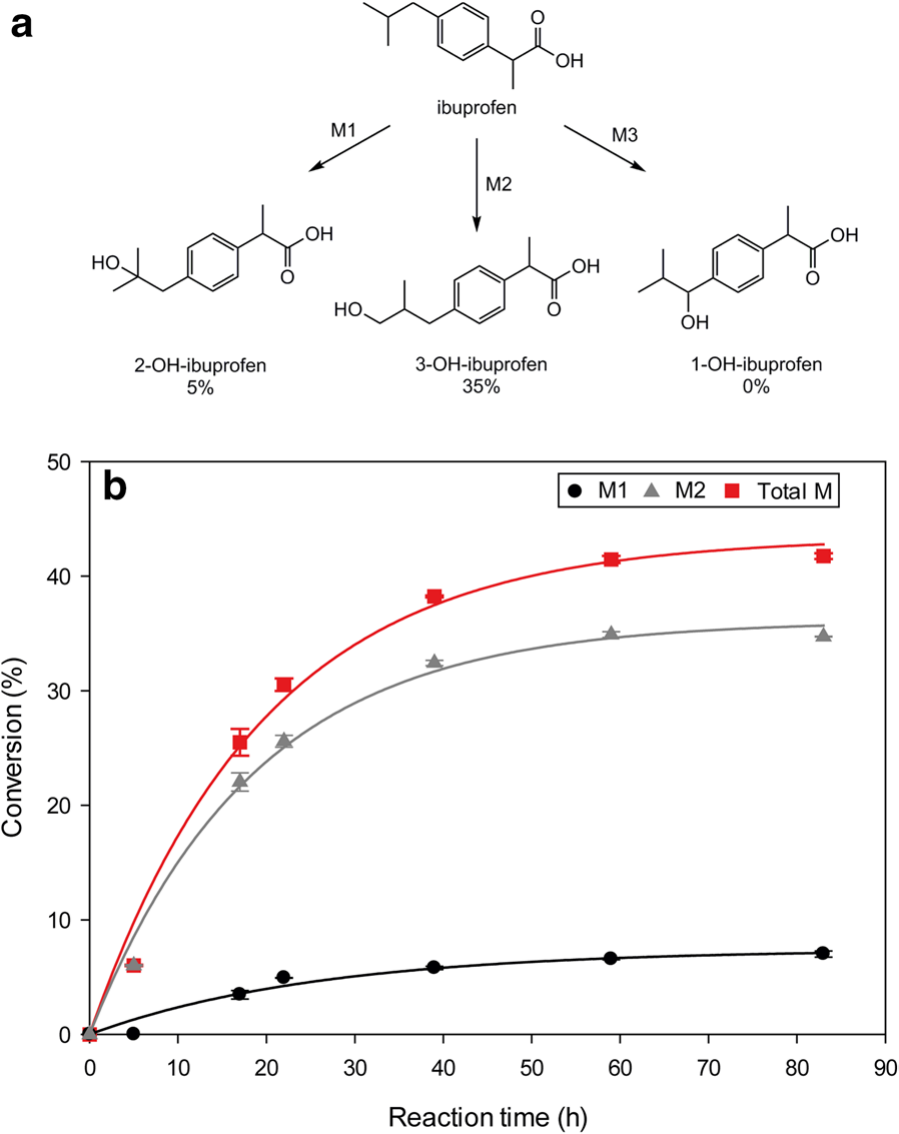
**a** Ibuprofen oxidation reactions catalysed by human CYPs. **b** Preparative scale ibuprofen oxidation by *P. pastoris* CYP2C9/CPR whole cells biocatalyst. Substrate concentration: 2 mM, reaction volume: 500 mL

Biomass produced by controlled cultivation in bioreactors was applied to evaluate its activity for preparative biotransformation of ibuprofen to its respective CYP2C9 metabolites. During Phase I metabolism in the human liver the drug ibuprofen is mainly converted into 1-hydroxyibuprofen, 2-hydroxyibuprofen, and 3-hydroxyibuprofen [24]. CYP2C8 and CYP2C9 are known as the main enzymes responsible for its stereoselective hydroxylation [50]. However, as reported by Neunzig et al*.*, CYP2C9 is the only known CYP able to produce relevant amounts of 3-OH-ibuprofen [24]. To evaluate the feasibility of a preparative scale production using *P. pastoris*, CYP2C9 expressing cells made by reliable cultivation in bioreactors were applied in a whole cell biotransformation approach using a reaction volume of 500 mL and an initial substrate concentration of 2 mM. Within 16 h a rather linear increase in conversion to 25% of total product was observed (Fig. 6), which further increased up to 40% within 86 h of reaction. As expected from small scale results (data not shown), metabolite 2 (35%), which was identified as 3-OH-ibuprofen by NMR spectroscopy (Additional file 2), was mainly produced, while metabolite 1 was only found to an extent of 5% (2-OH-ibuprofen). No relevant amounts of metabolite 3 could be detected (1-OH-ibuprofen), which was observed as a typical product in previous experiments employing a fungal bifunctional P450 homologue of the bacterial CYP102 [6].

A total product concentration of 0.8 mM was obtained starting with 2 mM of substrate ibuprofen with 0.70 mM of 3-OH-ibu and 0.1 mM of 2-OH-ibu, respectively as products and no 1-OH-ibuprofen. These results confirmed the potential of recombinant CYP2C9 catalysed biotransformations using *P. pastoris* whole cells as biocatalyst. In terms of space–time yield and specific production rate, values obtained with the *P. pastoris* whole cell biocatalyst were significantly higher—up to 55% and 21%, respectively—than the reported in the literature, in which the same proteins were expressed in a fission yeast system [24]. Moreover, the ratios between the two products (M2/M1) were in average around 5, and therefore, even higher than previous published values [24], confirming the high selectivity of this biotransformation for the 3-OH-metabolite. The main reaction parameters are presented in Table 2.

**Table 2:** Main parameters obtained for the preparative scale conversion of ibuprofen

Reaction time (h)	Average substrate conversion (%)	Product formed (mM)		Space–time yield (µmol product L^−1^ d^−1^)		Specific prod. rate (µmol product g whole cells^−1^ d^−1^)		Yield (µmol product g whole cells^−1^)	
		M1	M2	M1	M2	M1	M2	M1	M2
86	41.7	0.14	0.69	39	194	1.22	6.06	4.39	21.7

Despite the promising results obtained in the proof-of-concept ibuprofen biotransformation, the biocatalytic reaction could be further improved by addressing the impact of some important parameters such as dissolved oxygen availability, efficiency of NADPH regeneration system and/or heme group generation.

## Conclusions

Recombinant production of hCYPs has been intensively investigated and it is expected to continue attracting high attention by the pharmaceutical and chemical industry. Especially, a fast track from catalyst design to biocatalyst manufacturing and scalable biotransformation is essential to react on urgent and sometimes unexpectedly arising needs in drug development. In this work, a whole scalable bioprocess development for fine-tuned co-expression of CYP2C9 and CPR in the broadly available and efficient expression host *P. pastoris* has been successfully attained. To achieve this objective, an innovative bidirectional expression system using a small representative bidirectional promoter library with different regulation patterns was successfully implemented to achieve an optimized fine-tuned co-expression clone for the hCYP29 and its natural redox partner hCPR. Furthermore, for the first time, functionally active *P. pastoris* CYP2C9 whole cell biocatalysts were obtained from reliable and optimized bioreactor cultures, which finally were successfully tested for a biotransformation of interest based on the oxidation of ibuprofen. The biocatalyst used in the preparative test could convert more than 40% of the added substrate in a simple batch biotransformation and, more importantly, it demonstrated high selectivity for the product hydroxylated at position three.

Moreover, a deep study of using CYP2C9/CPR expressing *P. pastoris* whole cells focused on the production kinetics has been carried at bench top bioreactor scale. It has been demonstrated that the *µ* has a positive effect on *q*_*p*_, demonstrating that the production is coupled with the cell growth. Therefore, selecting a high *µ* in the bioprocess is advantageous to maximize *q*_*p*_ on methanol-free strategies. However, if the objective is to maximise the *Y*_*P/X*_ and, therefore, the biocatalyst activity, a bioprocess using mixed substrates based on the co-feeding of glycerol and methanol should be recommended. In these strategies, *µ* is decreased to the lowest value tested (0.05 h^−1^) to promote the methanol consumption.

Altogether, it is expected that this work will become a model, not only for the targeted fast development of new human CYP catalysts, but also as applicable reliable bioprocesses development for respective whole cell biocatalyst production and the respective catalysed biotransformations employing recombinant human CYPs expressed by *P. pastoris.* In a similar fashion, other biocatalysts relying on optimized co-expression rations can be developed. This progress will support fast and efficient drug development pipelines, which become especially challenging and important in case of pandemic diseases, where new drug candidates need to be advanced to the next phase within very short timelines.

## Materials and methods

### Strain generation

*Escherichia coli*/*P. pastoris* shuttle vectors were based on plasmid pPpT4 [51] and adapted as entry vectors for quick and simple cloning of bidirectional promoters by bisy (bisy GmbH, Hofstaetten, Austria). Synthetic DNA sequences were for these human open reading frames were obtained from IDT (Integrated DNA Technologies Inc., Coralville, Iowa, USA). By using Gibson assembly [52], a test set of 7 bidirectional promoters obtained from bisy GmbH (P_*DAS1/DAS2*_, P_*DAS2/DAS1*_, P_*PDC/PDF*_, P_*PDF/PDC*_, P_*DAS1-D6/DAS2-D8*_, P_*DAS2-D8/DAS1-D6*_, and P_*DAS2-699/DAS1-552*_) and described previously by Vogl et al*.* [30] to drive expression of full length hCYP2C9 (AL359672) including its hydrophobic N-terminal sequence and hCPR (accession AAH34277.1) were generated using the mentioned cloning plasmids. These cloning plasmids were transformed into the *P. pastoris* strain BSYBG11 Mut^S^ (Δ*aox1*, Mut^S^), which presents a slow methanol utilisation phenotype, which was obtained from Bisy GmbH. Generation of competent *P. pastoris* cells and transformation were performed according to Lin-Cereghino et al*.* [53]. In this strain generation the target expression cassette is integrated into the *Pichia* genome by homologous recombination. Due to the *Pichia* clonal variability, High Throughput Screening (HTS) were used for the further identification of the catalyst with the highest activity.

### Whole cell cultivation

#### High-throughput small scale cultures

Microscale 96 deep well cultivation was performed based on Weis et al*.* [41]. Single colonies were transferred to one well containing 250 µL of BMD1% (buffered minimal dextrose 1%) and cultivated for 60 h at 320 rpm and 28 °C. Subsequently, 250 µL BMM2% (buffered minimal methanol 1%) were added to induce gene expression. 8, 24 and 32 h after the first induction, 50 µL BMM10 (buffered minimal methanol 5%) were added. After 48 h of methanol induction, cells were harvested by centrifugation at 500 rpm. Cultivation in shake flask (Thomson 2 L ultra-yield flasks) was performed accordingly starting with 200 mL.

#### Chemostat cultures

Chemostat cultures were performed based on the methodology previously described by Garcia-Ortega et al*.* [48], but using glycerol as a substrate instead of glucose. In this case, the different cultures conditions compared were performed at a constant specific growth rate (*µ*) of 0.10 h^−1^, as intermediate value considering *µ*_*max*_ ≈ 0.20 h^−1^. All the operating parameters were kept constant as reported in the reference along the cultivations except the pH, which was controlled at different values of 5, 6 and 7.

#### Fed-batch cultures

Main details regarding medium composition, operational parameters and process strategies are described in previous works [45]. All the fed-batch cultures were based on a carbon-limited operational strategy using a pre-programmed exponential feeding profile of glycerol feeding solutions, which allows keeping a constant specific growth rate (*µ*) throughout the process, reaching a pseudo-stationary state. Upon co-substrate processes, two strategies were compared, one based on periodic methanol pulse additions and another in which the methanol concentration was kept constant at 3 g L^−1^ by means of a closed control loop. For this last case, the methanol concentration was determined by a Methanol Sensor System (Raven Biotech Inc., Vancouver, Canada), and controlled by performing a predictive-PI control strategy. Furthermore, off-line methanol determinations by HPLC were used to validate the control system. Further details about the methanol control system are described elsewhere [54, 55].

### Analytical methods

#### Biomass determination

Biomass concentration was measured by DCW using a protocol described elsewhere [56]. RSD was estimated to be under 3%. Biomass concentration was also measured by optical density at 600 nm using a DR3900 spectrophotometer (Hach Lange, Colorado, USA). RSD was estimated to be below 3%.

#### Carbon source and by-products quantification

The amounts of both carbon sources (glycerol, methanol) and potential fermentation by products were analysed by HPLC. The chromatography procedure and the column specifications were described elsewhere [57]. RSD was estimated to be lower than 1%.

### Whole cell bioconversions

#### High throughput screening (HTS)

Whole cell screenings using diclofenac as substrate were carried out in 96-well microtiter plates. After the harvest, cells were washed twice with 200 µL of assay buffer (100 mM KPi, pH 7.4) and then the biomass concentration was determined by optical density determination (OD_600_). Cells were frozen at − 20 °C for several days to obtain a better membrane permeability. Subsequently, 4 µL of a 100 mM diclofenac stock solution in methanol (MetOH) were added to each well and conversions were performed for 20 h at 28 °C and 320 rpm. Reactions were stopped by addition of 200 µL of MetOH/acetonitrile (1:1) and vigorously mixed. After centrifugation (10 min, 3200×*g*) 200 µL of supernatant were transferred into a fresh 96 well microtiter plate (polypropylene, V-shaped), sealed and analysed by HPLC–MS.

Fluorometric whole cell screenings based on the 7-methoxy-4-(trifluoromethyl)-2*H*-chromen-2-one (MFC) assay were performed using 95 μL of the cell suspension in assay solution using black microtiter plates. The reaction was started by the addition of 5 μL of 1 mM MFC to reach a final concentration 50 μM. Fluorescent metabolite formation was quantified measuring the fluorescence of the product 7-hydroxy-4-(trifluoromethyl)-2*H*-chromen-2-one (HFC). The fluorescence signal of the product formed (ex 410 nm/em 538 nm) was monitored every minute for a period of 1 h using a SynergyMX plate reader (BioTek Instruments Inc, USA). During the reaction the microtiter plates were kept at 30 °C.

#### Bioconversion reactor

For bioreactor samples, the enzymatic assays were performed in 50 mL falcon tubes. In this case, the washed cells were diluted to OD_600_ of 5 as a standard OD_600_ for all reactions. Then, 2 mL of cells were mixed with diclofenac to reach a 2 mM substrate concentration and reactions were carried at 28 °C and 200 rpm. After 20 h of incubation, 50 µL were taken and mixed with 450 µL of MetOH/acetonitrile (1:1), to stop the reaction and to extract both the substrate (diclofenac) and the product (4′OH-diclofenac). Both substrate and product concentrations were determined by HPLC–UV. Before its analysis, samples were centrifuged at 12000×*g* for 2 min and supernatants were filtered (0.45 µm).

The product-related results of the fermentations are presented in terms of activity units (AU). One AU is defined as the amount of enzyme which catalyses the production of 1 µmol of 4-hydroxy-diclofenac per hour under the assay conditions. Thus, this parameter provides an enzyme titer value in terms of (AU L^−1^).

### HPLC–UV/MS

HPLC/UV analysis were performed on a HPLC Dionex Ultimate 3000 (Dionex Corporation, Sunnyvale, CA, USA) series using a CORTECSTM C18 + 2.7 μm 4.6 × 150 mm column (Waters), at a temperature of 40 °C and a detector wavelength of 275 nm. The mobile phase was composed of Trifluoroacetic acid 0.1% (v/v) in water (Eluent A) and Trifluoroacetic acid 0.095% (v/v) in Acetonitrile 80% (Eluent B). Gradient-elution was performed at 0.7 mL min^−1^ as follows: 0.00–0.50 min: 95% A, 5% B; 0.50–13 min: 55% A, 45% B; 13.00–13.50 min: 20% A, 80% B; 13.5–15.00 min: 0% A, 100% B; 15.00–15.50 min: 0% A, 100% B; 15.50–20.00 min: 95% A, 5% B. Retention times were 12.04 for diclofenac and 8.22 4′-hydroxydiclofenac. For calculations, peak areas of the UV chromatograms recorded at 239 nm were used.

HPLC–MS was performed on a HPLC 1200 series (Agilent technologies, Santa Clara, CA, USA) equipped with an MSD SL detector with an electron spray ionization (ESI) unit as described previously [58]. A Kinetex® 2.6 μm C18 100 Å, LC Column 50 × 4.6 mm column (Phenomenex) was used. The mobile phase was composed of water (acidified with 0.1% formic acid) and acetonitrile (ACN). Gradient-elution was performed at 1.3 mL/min as follows: 0.00–1.00 min: 10% ACN; 1.00–3.50 min: 10–100% ACN; 3.50–4.00 min: 10% ACN. To allow a flow rate of 1.3 mL min^−1^, a splitter (2:1 ratio) was implemented in front of the ESI unit. 4′-hydroxydiclofenac (m/z 312) eluted after 2.2 min and diclofenac (m/z 296) after 2.4 min. For analysis of ibuprofen and its metabolites a stepwise gradient was used at 25 °C as described previously [6]: 0.00–1.00 min: 20% ACN; 1.00–3.00 min: 20–100% ACN; 3.00–5.00 min: 100% ACN; 5.00–6.00 min: 20% ACN re-equilibration. Metabolites were detected at 239 nm (VWD) and MS spectra were used to support data evaluation.

#### Preparative scale conversion of ibuprofen

Preparative scale conversions were performed as described previously [6, 58]. A total reaction volume of 500 mL including the whole cell catalysts corresponding to OD_600_ = 150 was used for oxidation of 206 mg ibuprofen dissolved in DMSO. Conversion was performed in a baffled 2 L shake flask over a period of 86 h. Samples of 200 µL were withdrawn at different times and analysed as described above.

### Nuclear magnetic resonance spectroscopy (NMR)

For recording NMR spectra, a Bruker Avance III 300 MHz FT NMR spectrometer with autosampler was used (300.36 MHz-H-NMR and 75.5 MHz-C-NMR). The residual protonated solvent signal serve as internal standard for interpretation of the chemical shifts δ (H-,C-NMR). To facilitate the interpretation, the C-spectra were proton decoupled to gain better identification of the peaks.

The chemical shift δ is indicated in ppm (parts per million) and the coupling constant J in Hz (Hertz). For the signal multiplicities the following abbreviations were most commonly used: s (singlet), bs (broad singlet), d (doublet), t (triplet), q (quadruplet), m (multiplet). Quaternary carbons are labelled as Cq.

## Supplementary Information

**Additional file 1: Figure S1.** Whole cell hydroxylation of diclofenac by *P. pastoris* CYP2C9/CPR whole cells cultivated respectively in bioreactor and in shake flask (UYF = ultra-yield flask). The wild type strain BSYBG11 was used as a negative control.

**Additional file 2.** Nuclear Magnetic Resonance Spectroscopy (NMR) analyses. **Figure S2A.** Product 1, ^1^H NMR (300 MHz, DMSO): δ 12.16 (bs, 1H); 7.15 (s, 4H); 3.96 (bs, 1H); 3.62 (q, *J* = 7.0 Hz, 1H); 2.61 (s, 2H); 1.31 (d, *J* = 14.4 Hz, 3H); 1.05 (s, 6H) ppm. **Figure S2B.** Product 1, ^13^C NMR (75.5 MHz, DMSO): δ 175.5; 138.5; 137.4; 130.4; 126.6; 69.3; 49.0; 44.3; 29.2; 18.5 ppm. **Figure S2C.** Product 2, ^1^H NMR (300 MHz, DMSO): δ 12.25 (s, 1H); 7.24–7.07 (m, 4H); 4.51 (s, 1H); 3.62 (dd, *J* = 13.8; 6.8 Hz; 1H); 3.23 (dd, *J* = 15.1; 8.3 Hz, 2H); 2.68 (dd, *J* = 13.2; 5.7 Hz, 1H); 2.25 (dd, *J* = 13.1; 8.3 Hz, 1H); 1.76 (dd, *J* = 12.9; 6.5 Hz, 1H); 1.34 (d, *J* = 7.0 Hz, 3H); 0.78 (d, *J* = 6.6 Hz, 3H) ppm. **Figure S2D.** Product 2, ^13^C NMR (75.5 MHz, DMSO): δ 175.5; 139.4, 138.5; 129.1; 127.2; 65.6; 44.3; 38.7; 37.6; 18.6; 16.5 ppm.

## Data Availability

The datasets used and/or analysed during the current study are available from the corresponding author on reasonable request.
