# Supplementary material for: Scalable production and application of Pichia pastoris whole cell catalysts expressing human cytochrome P450 2C9
Source: Microb Cell Fact. 2021 Apr 26;20:90. doi: 10.1186/s12934-021-01577-4 (PMC8074423; doi:10.1186/s12934-021-01577-4)
Supplement: Supplementary file 1 — Additional file 1: Figure S1. Whole cell hydroxylation of diclofenac by P. pastoris CYP2C9/CPR whole cells cultivated respectively in bioreactor and in shake flask (UYF = ultra-yield flask). The wild type strain BSYBG11 was used as a negative control. [file 12934_2021_1577_MOESM1_ESM.pdf]

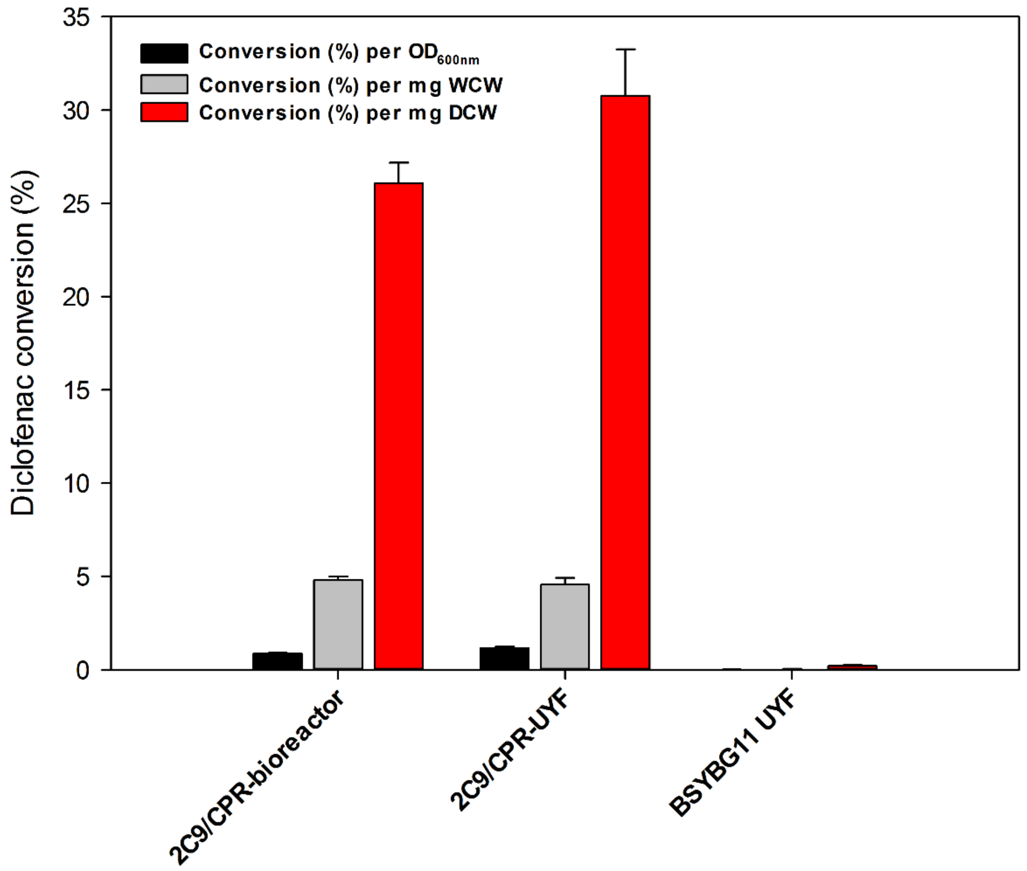

Figure S1. Whole cell hydroxylation of diclofenac by *P. pastoris* CYP2C9/CPR whole cells cultivated in bioreactor and in shake flask (UYF = ultra-yield flask) and the wild type strain (BSYBG11).
