# Supplementary material for: Scalable production and application of Pichia pastoris whole cell catalysts expressing human cytochrome P450 2C9
Source: Microb Cell Fact. 2021 Apr 26;20:90. doi: 10.1186/s12934-021-01577-4 (PMC8074423; doi:10.1186/s12934-021-01577-4)
Supplement: Supplementary file 2 — Additional file 2. Nuclear Magnetic Resonance Spectroscopy (NMR) analyses. Figure S2A. Product 1, 1H NMR (300 MHz, DMSO): δ 12.16 (bs, 1H); 7.15 (s, 4H); 3.96 (bs, 1H); 3.62 (q, J = 7.0 Hz, 1H); 2.61 (s, 2H); 1.31 (d, J = 14.4 Hz, 3H); 1.05 (s, 6H) ppm. Figure S2B. Product 1, 13C NMR (75.5 MHz, DMSO): δ 175.5; 138.5; 137.4; 130.4; 126.6; 69.3; 49.0; 44.3; 29.2; 18.5 ppm. Figure S2C. Product 2, 1H NMR (300 MHz, DMSO): δ 12.25 (s, 1H); 7.24–7.07 (m, 4H); 4.51 (s, 1H); 3.62 (dd, J = 13.8; 6.8 Hz; 1H); 3.23 (dd, J = 15.1; 8.3 Hz, 2H); 2.68 (dd, J = 13.2; 5.7 Hz, 1H); 2.25 (dd, J = 13.1; 8.3 Hz, 1H); 1.76 (dd, J = 12.9; 6.5 Hz, 1H); 1.34 (d, J = 7.0 Hz, 3H); 0.78 (d, J = 6.6 Hz, 3H) ppm. Figure S2D. Product 2, 13C NMR (75.5 MHz, DMSO): δ 175.5; 139.4, 138.5; 129.1; 127.2; 65.6; 44.3; 38.7; 37.6; 18.6; 16.5 ppm. [file 12934_2021_1577_MOESM2_ESM.pdf]

Nuclear Magnetic Resonance Spectroscopy (NMR)

For recording NMR spectra a Bruker Avance III 300 MHz FT NMR spectrometer with autosampler was used (300.36 MHz-H-NMR and 75.5 MHz-C-NMR). The residual protonated solvent signal serve as internal standard for interpretation of the chemical shifts δ (H-,C-NMR). To facilitate the interpretation, the C-spectra were proton decoupled to gain better identification of the peaks.

The chemical shift δ is indicated in ppm (parts per million) and the coupling constant *J* in Hz (Hertz). For the signal multiplicities the following abbreviations were most commonly used: s (singlet), bs (broad singlet), d (doublet), t (triplet), q (quadruplet), m (multiplet). Quarternary carbons are labeled as Cq.

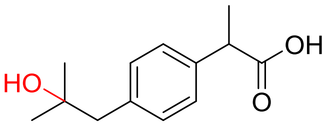

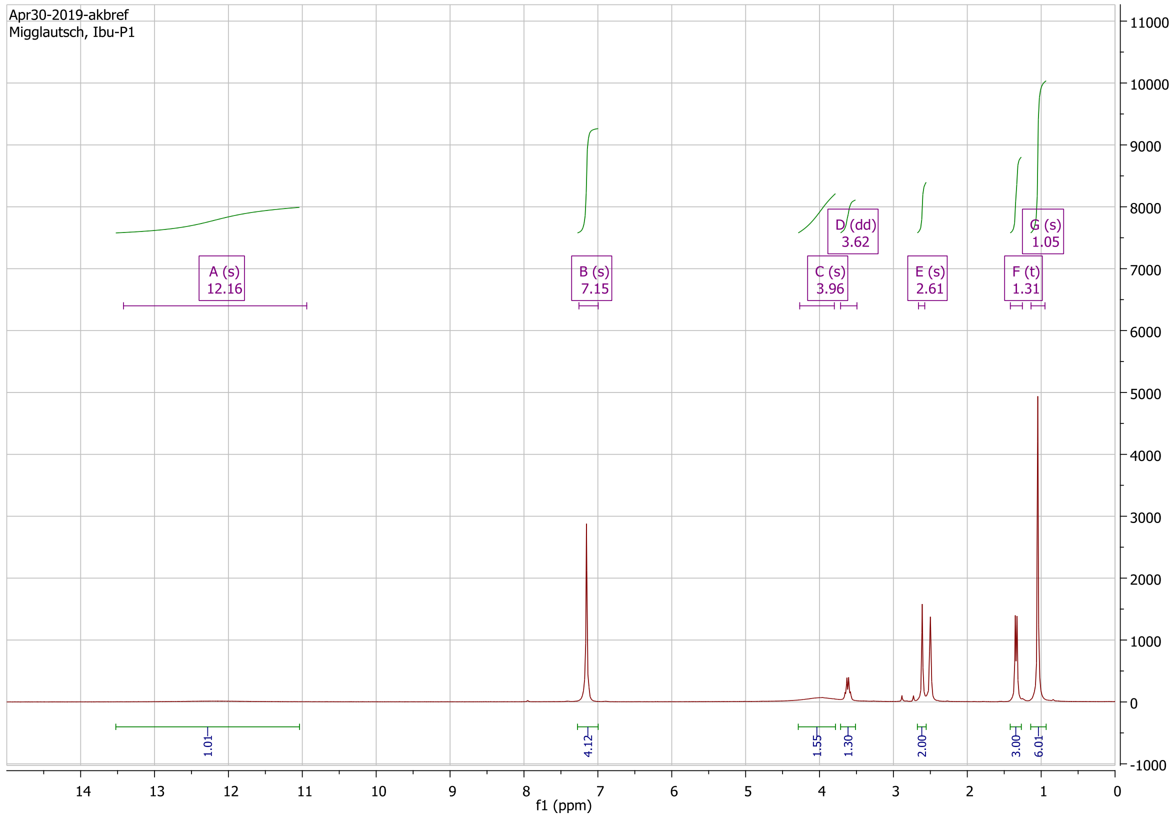

Figure S2A. Product 1. ^{1}H NMR (300 MHz, DMSO): δ 12.16 (bs, 1H), 7.15 (s, 4H), 3.96 (bs, 1H), 3.62 (q, *J* = 7.0 Hz, 1H), 2.61 (s, 2H), 1.31 (d, *J* = 14.4 Hz, 3H), 1.05 (s, 6H) ppm.

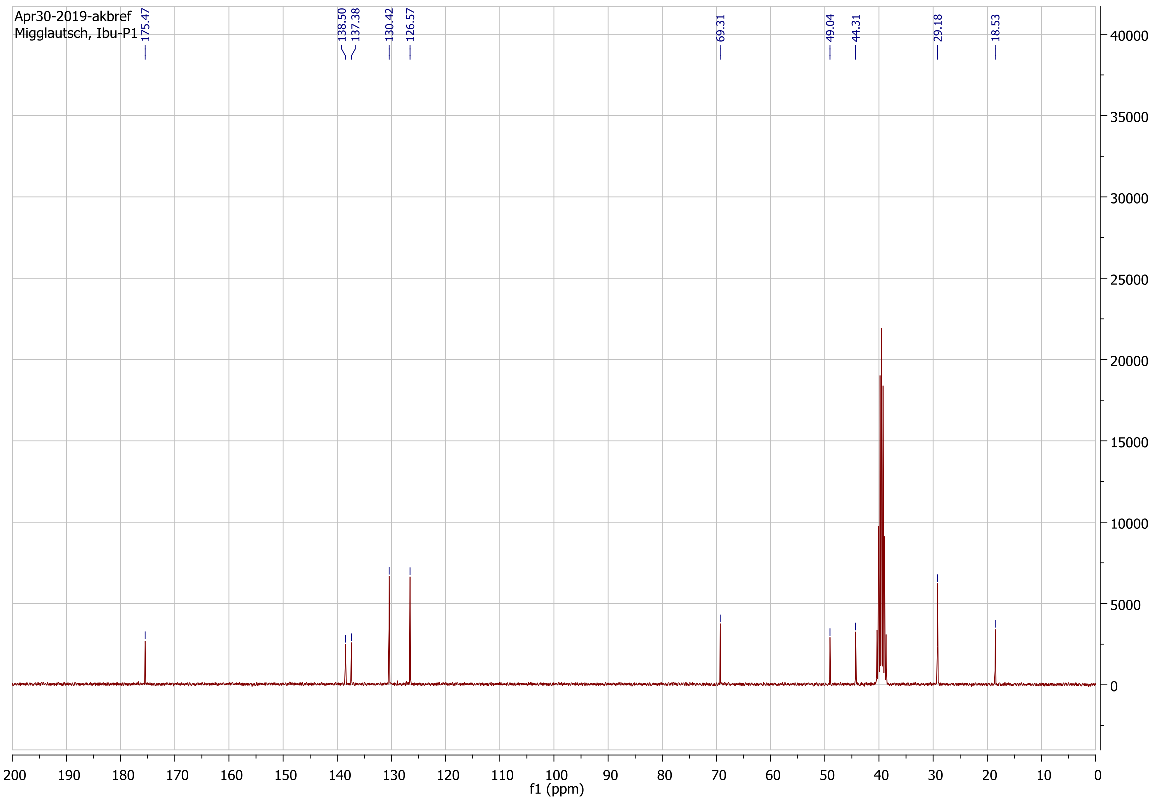

Figure S2B. Product 1. ^{13}C NMR (75.5 MHz, DMSO): δ 175.5, 138.5, 137.4, 130.4, 126.6, 69.3, 49.0, 44.3, 29.2, 18.5 ppm.

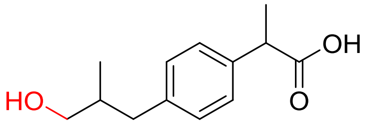

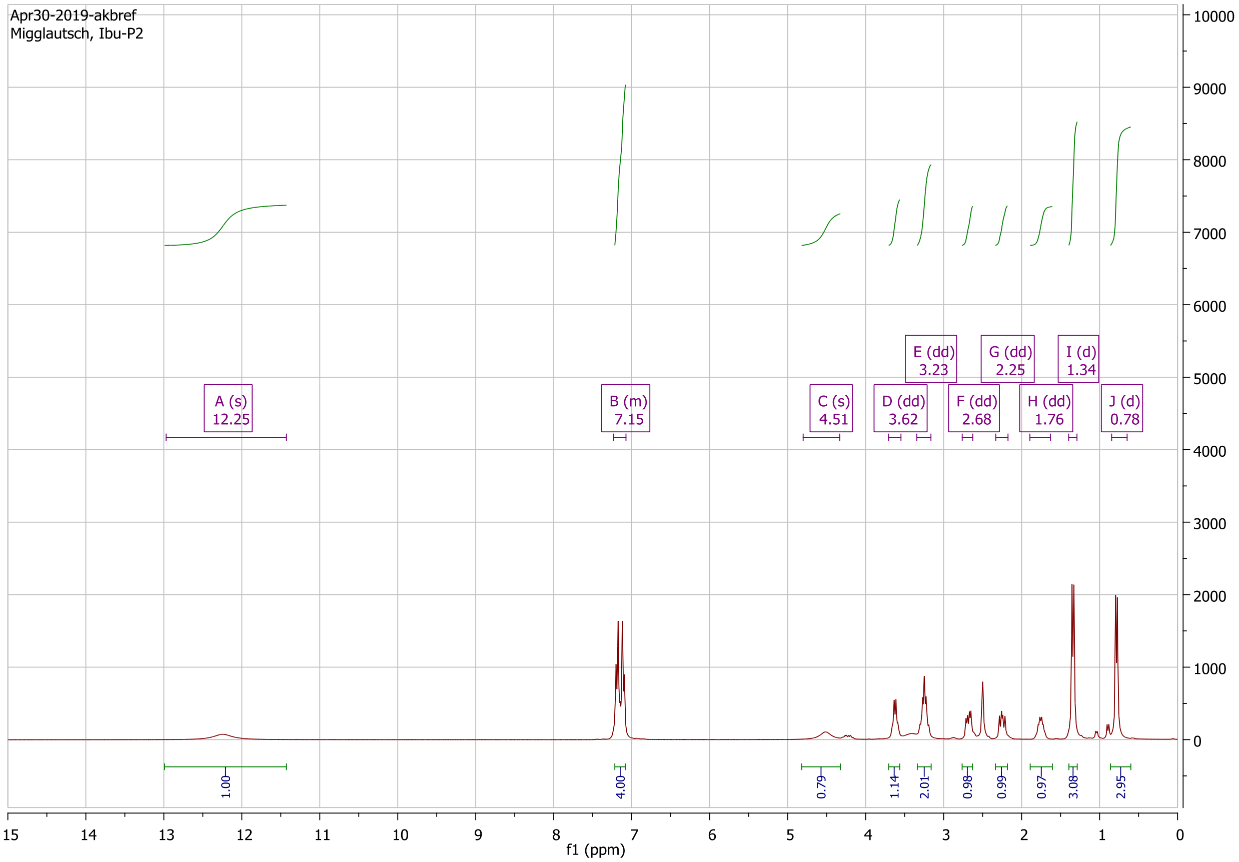

Figure S2C. Product 2. ^{1}H NMR (300 MHz, DMSO): δ 12.25 (s, 1H), 7.24 – 7.07 (m, 4H), 4.51 (s, 1H), 3.62 (dd, J = 13.8, 6.8 Hz, 1H), 3.23 (dd, J = 15.1, 8.3 Hz, 2H), 2.68 (dd, J = 13.2, 5.7 Hz, 1H), 2.25 (dd, J = 13.1, 8.3 Hz, 1H), 1.76 (dd, J = 12.9, 6.5 Hz, 1H), 1.34 (d, J = 7.0 Hz, 3H), 0.78 (d, J = 6.6 Hz, 3H) ppm.

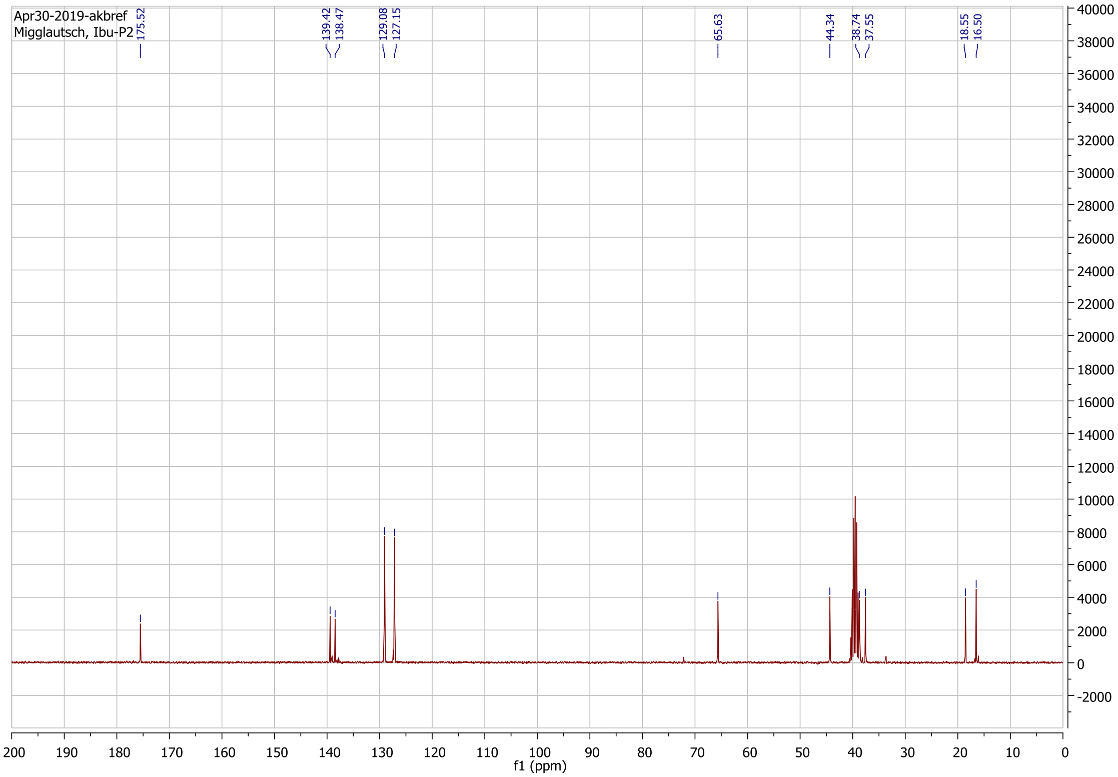

Figure S2D. Product 2. ^{13}C NMR (75.5 MHz, DMSO): δ 175.5, 139.4, 138.5, 129.1, 127.2, 65.6, 44.3, 38.7, 37.6, 18.6, 16.5 ppm.
